# Cost of Serving Others: A Moderated Mediation Model of OCB, Ego Depletion, and Service Sabotage

**DOI:** 10.3389/fpsyg.2021.595995

**Published:** 2021-03-15

**Authors:** Li Hongbo, Muhammad Waqas, Hussain Tariq, Farzan Yahya, Joseph Marfoh, Ahsan Ali, Syed Muhammad Ali

**Affiliations:** ^1^School of Management, Jiangsu University, Zhenjiang, China; ^2^NUST Business School, National University of Sciences and Technology, Islamabad, Pakistan; ^3^Department of Business Administration, Institute of Southern Punjab, Multan, Pakistan; ^4^School of Economics and Management, Tongji University, Shanghai, China; ^5^School of Management, Hefei University of Technology (HFUT), Hefei, China; ^6^Department of Engineering Management, National University of Sciences and Technology (NUST), Islamabad, Pakistan

**Keywords:** organizational citizenship behavior, ego depletion, service sabotage, psychological capital, Chinese service industry

## Abstract

Taking support from ego-depletion theory, this study examines ego depletion as a mechanism that explains how employees’ organizational citizenship behavior (OCB) leads to antagonistic consequences, i.e., service sabotage. Employees’ positive psychological capital (PsyCap) is considered a moderator. PROCESS macro was used to test all the hypotheses using time-lagged, dyadic data collected from 420 employees and their 112 their supervisors associated with the service industry in China. This study finds that employees’ exhibition of OCB is positively linked to ego depletion, which in turn drives service sabotage behavior. Furthermore, employees’ PsyCap weakens the effect of OCB on employees’ ego depletion. This study highlights the dark side of OCB, the mechanism through which it causes adverse effects, and the moderating effect of PsyCap. It also provides insights to the organizations for managing service sector employees to effectively interact with customers.

## Introduction

Employees’ behavior is crucial for organizational success. However, this phenomenon is more essential in the service sector where frontline employees’ behavior is considered a key to achieving customers’ satisfaction and even organizational survival in the long run (Harris and Ogbonna, 2012). Despite the well-accepted importance of employees’ behavior for organizational success, it is quite astonishing that many employees exhibit such dysfunctional behavior that is detrimental to organizations. For instance, Harper (1990) estimated that 75% of employees had been perpetrators in service sabotage, while Harris and Ogbonna (2002) reported an incidence rate of 85%. Since customer loyalty is undermined, service sabotage can exert long-term, detrimental effects on the reputational and financial capital of organizations (Hongbo et al., 2019).

The absence of focus on the sabotage issue was first identified around 30 years ago by Jermier (1988, p. 30), when he noted that “well-developed concepts of workplace sabotage are not incorporated into organizational social sciences, leaving its meaning, causes, and consequences subject to folk wisdom, popular opinion, and casual conjecture.” Although there are some studies (Skarlicki et al., 2008; Yeh, 2015) of service sabotage, these have been plagued by some limitations (Harris and Ogbonna, 2012). For instance, the limited studies on sabotage have tended to be externally driven, while reasons to sabotage the service do not need to be always an external factor. We argue that systematic empirical research to highlight organizational internal factors that cause employees’ service sabotage behavior is important for several reasons. Firstly, the growing service sector is now a key source of employment and economic activity in many industrialized economies. For that, a greater comprehension of the range of employees’ behavior which impacts organizational success is required. Secondly, as reported, service sabotage behavior costs United States firms up to $200 billion per year (Harris and Ogbonna, 2012; Lee and Ok, 2014); this far-reaching, detrimental impact of service sabotage behavior has prompted interest on the part of researchers to identify and investigate the antecedents of sabotage behavior. Specifically, an exploration of organizational internal factors that cause service sabotage behavior is relatively ignored in the literature. To the best of our knowledge, most of the studies conducted so far have considered customer negative events as the primary reason for service sabotage (Hongbo et al., 2019). To further extend the literature, the study aims to present an empirical insight into how organizational citizenship behavior (OCB), an organizational internal factor, causes employees’ service sabotage behavior.

Organizational citizenship behavior, defined as “individual’s behavior that is discretionary, not recognized by formal reward system and, in the aggregate, one that promotes the efficient and effective functioning of the organization” (Babcock-Roberson and Strickland, 2010, p. 317), is considered one of the essential requirements for organizations to effectively operate and achieve their desired goals (Organ, 1990). Most of the research (Indarti et al., 2017; Lavy and Littman-Ovadia, 2017) conducted so far has identified several positive outcomes associated with OCB for its recipients and organizations, what is abstruse, however, is the cost associated with OCB for those who exhibit such behavior (Bolino and Turnley, 2005; Deery et al., 2017). For instance, it has been shown to positively impact employee performance and well-being, which, in turn, has salient follow-on effects on the organization (Lee and Allen, 2002; LePine et al., 2002). Understanding how OCB affects its actors is an important issue that has the potential to expand the theory and practices. As mentioned, OCB refers to anything that employees choose to do, of their interest, which often lies outside of their specified contractual obligations. Given this, it is quite possible that exhibiting OCB may cause personal costs for individuals. Supporting the notion, the ego-depletion theory (Baumeister et al., 1998) also suggested that regulatory resources are depleted in performing tasks which later disturb individuals’ normal functioning. Ego depletion originally came out of social psychology. It was still applied to and investigated in various fields, such as personality, consumer behavior, decision making, cognitive psychology, and organizational behavior (Friese et al., 2019; Dang et al., 2020). It refers to the situation when individuals perform poorer on self-control task after having already engaged in a previous task requiring self-control. As the theory suggests, when resources are depleted, future regulatory efforts become increasingly difficult. Thus, the greater the regulatory activities employees perform throughout their day, the more difficult it becomes for them to maintain effort, persistence, and ultimately adequate performance on a host of tasks. Also, depleted employees are most likely to cheat and misinterpret organization performance, deceive and undermine others, and are verbally abusive toward peers, supervisors, subordinates (Barnes et al., 2015). With this, our study proposes that the exhibition of OCB causes personal cost, i.e., ego depletion, which restricts employees to maintain their service delivery standards to their customers; thus, they sabotage their services.

From the above discussion, a potential paradox emerges. On the one side, exhibiting OCB is desired and promoted in organizations. On the other side, there are also some costs associated with OCB. However, this condition does not apply to all individuals. Individuals indeed differ in their personalities; they behave differently in different situations. For that, they may not equally experience costs or benefits after the exhibition of OCB. This study considers positive psychological capital (PsyCap), an individual’s positive psychological state of development, as an individuals’ difference and examines what those high in PsyCap experience after the OCB exhibition. This study contributes to the existing literature in three different ways. Firstly, the limited but burgeoning literature on service sabotage mainly relies on external factors for employees’ service sabotage behavior.

**FIGURE 1:**
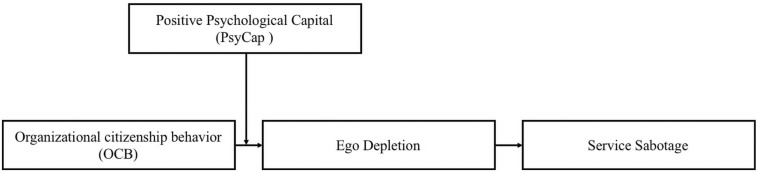
The proposed moderated mediation model.

In contrast, this study integrates ego-depletion theory with research on service sabotage to examine how those employees’ who exhibit OCB behave with their customers when their resources are depleted. Secondly, grounding on ego-depletion theory, we posit and test the idea to explain how one phenomenon (OCB), which is highly desired in organizations, becomes costly (service sabotage) for the organizations. This study posits that the immediate effect of OCB is ego depletion, which results in a situation where employees may not properly perform as before and involve in dysfunctional behavior. Thirdly, this study considers PsyCap as an individual difference and suggests that it has important implications for the resource-based process. Explicitly, this study considers PsyCap a resource-providing factor and hypothesizes that individuals high in PsyCap are less likely to experience ego depletion and, consequently, to be less involved in sabotage behavior. Lastly, most of the previous studies on service sabotage are cross-sectional. Therefore, the present study aims to contribute to the existing literature on service sabotage by providing robust empirical evidence through a time-lagged and dyadic data set. Hence, grounding on ego-depletion theory, we first develop the hypotheses, i.e., positive association between OCB and service sabotage through mediating effect of ego depletion, which explains “how perceived OCB leads to service sabotage?” Next, we theorize the moderating role of PsyCap to answer “how individual differences could moderate the hypothesize relationships?” The broader contributions to theory, practical implications, and suggestions for future research are discussed. Our proposed moderated mediation model is depicted in Figure 1.

## Literature Review and Hypothesis Development

The lifeblood of a service organization is its employees, particularly frontline, customer contact employees. Unlike tangible products, service is produced and consumed at the same time, making the frontline service employee essentially the service producer. This aspect of services brings into sharp focus the vital role that service employees play in delivering high-quality services. However, although a substantial amount of service quality research has focused on customers’ perceived service quality, relatively little attention has been paid to explore which factors impact service employees’ behavior. Research has shown that the service sector employees’ behavior often contrasts with what is desired (Harris and Ogbonna, 2009, 2012). That is, employees habitually put up attitudes that retrogress service quality, i.e., *service sabotage*. Sabotage incidents are alarmingly common in the service sector (Kao et al., 2014) and can occur daily (Harris and Ogbonna, 2006). The consequences associated with such sabotage include a decline in customer satisfaction and perceived service quality, decreased customer loyalty, and lower customer commitment to the organization (Harris and Ogbonna, 2002, 2009). These detrimental effects on organizational profit, growth, and even survival have motivated the organizational researchers to identify such factors that instigate such dysfunctional behavior (Hongbo et al., 2019). Research published on the said issue is primarily based on external factors (e.g., customer mistreatment and customers’ negative events) as antecedents of service sabotage (Tao et al., 2019). Additionally, scholars (Kao et al., 2014; Lee and Ok, 2014) also highlighted some internal factors (such as emotional labor and workplace stressor) which motivate employees’ service sabotage behavior. This study aims to extend the research on organizational internal factors that cause service sabotage. For that, we took OCB as a resource depletion factor and propose that it results in service sabotage behavior.

Organizational citizenship behavior refers to a behavior that is not formally requested or directly rewarded but functional to an organization’s operations (Smith et al., 1983). It consists of interpersonal helping behaviors beyond one’s immediate role requirements (Bateman and Organ, 1983; Settoon and Mossholder, 2002). It is also considered one of the most fundamental requirements for organizations to effectively operate and succeed (Organ, 1997). Thus, employees must be willing to go beyond their job description’s enforceable requirements to contribute toward organizational success. The researchers have long acknowledged that doing something for the organization or others related to the organization beyond the assigned tasks is an important component for organizational success (Lee and Allen, 2002; LePine et al., 2002). For instance, having conscientiousness and helping others results in decreased inter-group conflict and allow managers to focus on more pressing matters. OCB has also been documented as an organizational motive to effectively and efficiently achieve organizational goals (Daniels et al., 2006). Exploring the effect of OCB in the service industry, Bell and Menguc (2002) suggested that OCB is positively associated with positive customer-oriented behavior. In another study, Bienstock et al. (2003) also found a positive relationship between OCB demonstrated by service employees and the extent to which service is delivered according to service standards (Morrison, 1996). Moreover, Gyekye and Haybatollahi (2015) documented a significant positive association between OCB and job satisfaction among 320 Ghanaian industrial workers.

It is quite possible that OCB may not always lead to positive outcomes; it may end up in some personal cost for its exhibitors. Bolino and Turnley (2003) highlighted that employees might experience escalating citizenship behavior when exhibiting OCB becomes so normative for them to be seen as going the extra mile. Besides, they also stated that it becomes harder for employees to get away from work and contribute to competition among workers to be seen as the most committed employees. Bergeron (2007) also supported the same fact and stated that the value of OCB decreases over time because the cost (energy and time) associated with performing OCB outweighs its benefits (reputation and rewards). Scholars claim that OCB has some negative personal implications for individuals who are involved in OCBs. For instance, an exhibition of OCB causes stress (Organ and Ryan, 1995). Supporting the idea, Bolino and Turnley (2005) found that employees who are engaged in individuals’ initiatives experience job stress and work–family conflict. While several studies have highlighted the personal cost of OCB, it has also been noted that OCB has professional costs. As Bergeron (2007) noted, OCB is not something that occurs in a vacuum; engaging in OCB may cause adverse effects on employees’ task performance, which consequently damages their career. Specifically, the more time employees spend on performing OCB, the less time they spend on task performance, which affects organizational performance in a broader sense. Taken together with the above discussion, this study assumes that there can be a negative personal outcome (ego depletion) which then causes an adverse organizational outcome (service sabotage) for employees who go the extra mile for organizations and for those within the organizations.

Ego depletion, here taken as the personal cost of OCB, is defined as “a temporary reduction in the self’s capacity or willingness to engage in volitional action (including controlling the environment, controlling the self, making choices, and initiating an action) caused by the prior exercise of volition” (Baumeister et al., 1998, p. 1253). The theory of ego depletion (Baumeister et al., 1998) refers to the idea that self-control or willpower draws upon a pool of individuals’ regulatory resources that are used up over time. Thus, people feel depleted when these regulatory resources are diminished owing to the performance of activities which require self-control. One of the most important and powerful features of human psychology is volition capacity, otherwise known as will power, including choice and self-regulation (Baumeister and Leary, 1995). People show a remarkable capacity to regulate the self and overcome the impulses and drives that tempt them to engage in violent actions when provoked or procrastinate when they are at work (Steel, 2007). The ability to attain deliberative control over impulses and abstain from gratifying immediate needs and desire is extremely adaptive. It enables people to engage in goal-directed behavior to bring about desirable long-term outcomes (Fishbach and Labroo, 2007). The literature (Bauer and Baumeister, 2011; Baumeister et al., 2018) suggests that self-regulatory operations consume resources that are depleted afterward. When resources (limited) deplete, individuals are subsequently less successful at responding actively, even in a seemingly unrelated sphere of activity. The implication is that some resources akin to energy or strength are expended in the regulatory process, creating a state that has been dubbed ego depletion. Once depleted, employees’ capacity to exert self-control or to exhibit appropriate behavior based on limited resources becomes difficult, thereby leading to inappropriate behavior on employees’ part (Yam et al., 2014). Similarly, OCB has been documented as a situation where employees perform beyond their assigned tasks, which surely consumes more regulatory resources, and hence leaves actors in a depleted state.

As explained, OCB causes personal cost in the form of ego depletion, resulting in adverse consequences at the organizational level, i.e., *service sabotage*. To support this notion, we borrow support from ego-depletion theory, which claims that depletion caused by initial exertion dampens employees’ control on subsequent tasks. The prior literature (Carter et al., 2015; Dang, 2018) noted that ego-depleted employees lack sufficient resources to perform aptly and maintain their prosocial and productive behavior. Ego depletion lessens employees’ ability to maintain their extra-role performance and adversely affects their assigned role performance. Specifically, this study posits that the immediate result of OCB is ego depletion, which results in a situation where employees are not as devoted as before and do not hold appropriate behavior toward others and sabotage the services. Specifically, we propose the following:

Hypothesis 1: The positive relationship between OCB and service sabotage is mediated by ego depletion.

### Moderating Effect of Positive PsyCap

Positive PsyCap is defined as “an individual’s positive psychological state of development that is characterized by, having confidence (self-efficacy) to take on and put in the necessary effort to succeed at challenging tasks; making a positive attribution (optimism) about succeeding now and in the future; persevering toward goals and, when necessary, redirecting paths to goals (hope) to succeed; and when beset by problems and adversity, sustaining and bouncing back and even beyond (resilience) to attain success” (Luthans et al., 2007, p. 3). Largely stimulated by the positive psychology movement, there has been a call to go beyond human capital by focusing on what has been termed “positive psychological capital” (Luthans et al., 2007). PsyCap is not only concerned with “who you are” (i.e., human capital) but also in the developmental sense “who you are becoming,” your “best self” (Luthans et al., 2007, p. 20).

Research indicates that PsyCap has implications for combating stress and facilitates positive organizational change (Avey et al., 2008), supportive organizational climate, and employees’ performance (Luthans et al., 2008). Researchers in the service industry have identified similar implications. For instance, Avey et al. (2010b) found a positive association between PsyCap and both the financial and manager-rated performance of employees in the service industry. Moreover, Karatepe and Karadas (2015) noted that PsyCap is positively linked with work engagement, job satisfaction, career satisfaction, and frontline employees’ life satisfaction in the hospitality industry. Given the general expectancy of success derived from optimism and the belief in personal abilities derived from efficacy, those high in PsyCap report higher job satisfaction, performance, and commitment in the United States’ insurance service firms (Luthans et al., 2008). The literature also suggests that PsyCap predicts employees’ innovative behavior (Jafri, 2012), lower turnover intention, and improved general well-being. A primary explanatory mechanism for the effect of PsyCap on employee attitudes is that those higher in PsyCap expect good things to happen at work (optimism). They believe that they create their success and are more impervious to setbacks than those lower in PsyCap. The high level of optimism regarding the future and confidence in their own ability to succeed in the current job motivates them to take charge of their destinies (Seligman, 2000), self-select into challenging endeavors (Bandura, 1997), engage the necessary efforts and resources, and persevere in the face of obstacles (Stajkovic and Luthans, 1998), rather than become “quitters.”

In addition to desirable attitudes, research has found PsyCap to be negatively related to undesirable employee attitudes, such as cynicism toward change or turnover intentions (Avey et al., 2011). Specifically, based on the optimistic expectancies of future events and resilience to setbacks, those high in PsyCap reported being more open and less cynical about change in their organization (Avey et al., 2010a). Moreover, Bakker and Demerouti (2007) argue that holding job and personal resources constant, job demands create distress on employees, leading to psychological exhaustion, anxiety, and impaired health. However, positive psychological resources, such as efficacy and optimism, counteract the distress from these demands, such that the components of PsyCap act as a suppressor of stress and anxiety. Taking support from prior literature, this study assumes that individuals high in PsyCap have positive expectations about future outcomes and greater belief in their ability to deal with various challenges involved in the job. These positive psychological states motivate individuals to exert greater effort and perform well in their job. For that, we propose that PsyCap moderates the adverse effects of OCB; specifically, we propose that PsyCap weakens the positive association between OCB, ego depletion, and service sabotage.

Hypothesis 2(a): The positive relationship between OCB and ego depletion is moderated by PsyCap, such that this association is weaker when PsyCap is high.

Hypothesis 2(b): The indirect positive relationship between OCB and service sabotage through ego depletion is moderated by PsyCap, such that the mediated relationship is weaker when PsyCap is high.

## Materials and Methods

### Sample and Procedure

The multi-source and multi-wave data were collected from individuals working at hair salons in Jiangsu province, China, to the formal moderated mediation model. Research assistants were hired to personally visit the salons and collect the required information. To qualify for the sample, individuals were required to be full-time working adults with a direct supervisor. The survey was conducted in three stages, and the time lag was 2 weeks. All participants were voluntary and received our promise of confidentiality. To encourage maximum participation, we also offered gifts to the participants, for example, coffee or movie coupons. Three different surveys were designed. Survey 1 was designed for supervisors to report their subordinates’ OCB. Survey 2 was designed to collect employees’ demographics, control variables, ego depletion, and positive PsyCap. Survey 3 was designed for supervisors to rate their subordinates’ service sabotage behavior. In Stage 1, research assistants formally invited the direct supervisors and requested them to nominate their full-time working employees. Each direct supervisor was asked to complete Survey 1. In Stage 2, 2 weeks after Stage 1, we contacted the direct supervisors’ who nominated employees to provide the rating of Survey 2. In Stage 3, 2 weeks after Stage 2, we requested the direct supervisors (who provided the rating of Survey 1) to rate Survey 3. The surveys were matched by the name of the employee mentioned in each survey. Scales developed in English were used in designing the surveys, so we translated them into Chinese. Two Chinese bilingual academicians lend their expertise to confirm the back-translation’s quality and accuracy (Brislin, 1980).

At Stage 1, research assistants contacted 191 direct supervisors to complete Survey 1 and to provide their subordinates’ information. We received the complete ratings of 129 direct supervisors, and they also provided the information of 523 subordinates. At Stage 2, we contacted the 523 subordinates to provide ratings of Survey 2. We received a complete rating of 483 subordinates. At Stage 3, we contacted the 129 direct supervisors to complete Survey 3. Finally, we collected 420 responses from 112 direct supervisors and their 420 subordinates following the match survey procedure.

### Measures

#### OCB

We followed Babcock-Roberson and Strickland (2010) to measure OCB by using a 24-item scale from Smith et al. (1983). The sample items include “I help others who have been absent” and “I attend function not required but that help my company image.” Responses were measured on a 5-point (1 = strongly disagree, 5 = strongly agree) Likert scale.

#### Ego Depletion

Following Lanaj et al. (2016), we measured ego depletion by using a five-item scale developed by Twenge et al. (2004). The sample items include questions like “Generally speaking, I feel like my willpower is gone” and “I feel drained.” The responses were also recorded on a 5-point (1 = not at all, 5 = very much) Likert scale.

#### Positive PsyCap

Following the other studies (Avey et al., 2010a; Luthans et al., 2010), PsyCap was measured by using the 24-item scale from Luthans et al. (2007a). The 24-item scale consists of four dimensions, hope, resilience, optimism, and efficacy, each with a six-item scale. The sample items include “If I should find myself in a jam at work, I could think of many ways to get out of it,” “I always look on the bright side of things regarding my job,” and “I usually manage difficulties one way or another at work.” The responses were reported on a 5-point (1 = strongly disagree, 5 = strongly agree) Likert scale.

#### Service Sabotage

Following Hongbo et al. (2019), service sabotage was measured by the three-item scale developed by Chi et al. (2013). The supervisors were requested to report their employees’ sabotage behavior on a 5-point (1 = never, 5 = always) Likert scale. The sample items include “He/she deliberately mistreats his/her customers” and “He/she behave negatively toward customers.”

#### Control Variables

Following prior literature (Duffy et al., 2012; Hongbo et al., 2019), we controlled for demographic variables including supervisor’s and subordinate’s gender, supervisor’s and subordinate’s age, subordinate’s education, and experience in the same industry. Besides, as the research documented (Chi et al., 2013) customer negative events as a major reason for employees’ service sabotage behavior, we also controlled it. The three-item scale measured customer negative events by Chi et al. (2013). The sample items include “Customers made unreasonable demands that I could not fulfill,” and the responses were recorded on a 5-point (1 = strongly disagree, 5 = strongly agree) Likert scale.

### Analytical Approach

The participants worked independently of one another and came from different hair salon outlets, but many employees worked under the same supervisor and reported them. Therefore, it leads to the notion that employees’ rating could not be independent of each other and might potentially violate the assumption of ordinary least squares (OLS) regression. This violation could result in a biased estimate of standard errors and invalid test statistics. To address this concern, there is a need to analyze the data at the individual and supervisor levels (i.e., analyzing data at two levels). Thus, we computed the intraclass coefficient 1 and the intraclass coefficient 2 to determine the appropriate analysis level. ICC1 represents the amount of variance that resides between supervisors for each study variable, whereas ICC2 represents the supervisor’s stability for each study variable. The ICC1 for OCB, ego depletion, service sabotage, and PsyCap were 0.08, 0.08, 0.11, and 0.23, respectively. In line with this, the ICC2 for OCB, ego depletion, service sabotage, and PsyCap were 0.09, 0.10, 0.28, and 0.51, respectively. All of these values are below the generally accepted level of 0.70. These values suggest insufficient variance between supervisors coupled with low stabilities of their means to warrant using a multi-level approach.

Moreover, we followed the recent studies (e.g., Hongbo et al., 2020; Latif et al., 2020; Weng et al., 2020) and calculated a corrected (unbiased) *F* statistic for each of our analyses (Kenny, 1995) as this is a more conservative test. Results indicated that all of our corrected *F* statistics remained significant and decreased by no more than 0.10. All of these results, coupled with Kenny’s (1995) statement that if ICC1s are below 0.3, it is relatively safe to analyze data at the individual level, lead us to the decision to analyze all variables in the study at the individual level.

By doing so, we measured all the variables of our study at the individual level. We requested direct supervisors to rate their subordinates’ OCB (e.g., “he/she [subordinate name] help others who have been absent” and “he/she [subordinate name] attend function not required but that help his/her company image”) and subordinates’ service sabotage behavior (e.g., “he/she [subordinate name] deliberately mistreats his/her customers” and “he/she [subordinate name] behave negatively toward customers”). Following this, we also requested subordinates to rate their ego depletion (e.g., “generally speaking, I [subordinate name] feel like my willpower is gone” and “I [subordinate name] feel drained”) and positive psychological capital (e.g., “if I [subordinate name] should find myself in a jam at work, I [subordinate name] could think of many ways to get out of it,” “I [subordinate name] always look on the bright side of things regarding my job,” “I [subordinate name] usually manage difficulties one way or another at work”).

We followed the recent studies (e.g., see Eissa and Lester, 2017; Hongbo et al., 2019; Lee et al., 2019) to conduct data analysis through SPSS PROCESS macro developed by Preacher et al. (2007). Our study developed formal mediation and moderated mediation (two-way interaction) hypotheses, so we performed a series of analyses described by Preacher et al. (2007). First, the Hayes PROCESS macro (Hayes, 2013) allowed us to obtain a bias-corrected confidence interval (CI) using bootstrapping (using 10,000 bootstrap samples) to establish the significance of the mediation. Also, the Hayes PROCESS macro applied the Sobel test (normal theory approach) to test the formal mediation hypothesis. Second, the Hayes PROCESS macro also allowed us to test the moderated mediation model’s index and the conditional indirect effects of OCB on service sabotage via ego depletion at different values of PsyCap (−1 SD, M, and + 1 SD). We also applied the approach of Edwards and Lambert (2007) along with the procedure of the Hayes PROCESS macro to conduct a simple slop test and plot a graph to test the moderated mediation model (two-way interaction). Thus, we first used the SPSS “PROCESS macro” Model 4 to test our formal mediation hypothesis (i.e., Hypothesis 1). We then utilized the SPSS “PROCESS macro” Model 8 to test our proposed moderated mediation model (i.e., Hypotheses 2a and 2b).

## Results

### Descriptive Statistics

Table 1 provides our study’s descriptive statistics (e.g., standard deviations, means, and estimated coefficient alpha values) and intercorrelations. As anticipated, the preliminary analyses support our hypotheses. OCB is positively related to ego depletion (*r* = 0.36, *p* < 0.01) and service sabotage (*r* = 0.26, *p* < 0.01). Ego depletion is also positively related to service sabotage (*r* = 0.31, *p* < 0.01). Moreover, PsyCap is positively related to OCB (*r* = 0.39, *p* < 0.01) but negatively related to ego depletion (*r* = −0.31, *p* < 0.01) and service sabotage (*r* = −0.32, *p* < 0.01).

**TABLE 1:** Intercorrelations, descriptive statistics, and estimated reliabilities among the variables.

Variables	M	SD	1	2	3	4	5	6	7	8	9	10	11
Supervisor Gender^1^	1.62	0.46	(–)										
Supervisor Age^2^	2.31	0.77	–0.08	(–)									
Subordinate Gender^3^	1.47	0.50	–0.10	0.04	(–)								
Subordinate Age^4^	2.58	1.13	–0.02	0.06	0.01	(–)							
Subordinate Education^5^	2.81	1.01	0.10	0.10	–0.01	0.04	(–)						
Subordinate Experience^6^	3.41	1.22	0.09	–0.03	–0.09	0.06	−0.15*	(–)					
Customers’ Negative Events	2.99	0.89	–0.06	0.05	0.04	–0.02	0.01	0.04	(0.91)				
OCB	3.80	0.76	–0.01	–0.03	0.02	–0.08	–0.02	–0.01	0.17*	(0.87)			
Ego Depletion	4.29	0.51	–0.11	0.07	0.10	–0.01	–0.01	−0.16*	0.10	0.36**	(0.79)		
Service Sabotage	4.36	0.43	0.09	0.03	–0.08	0.12	–0.02	0.01	–0.03	0.26**	0.31**	(0.82)	
PsyCap	3.21	0.88	0.07	–0.06	0.05	–0.10	0.04	0.04	–0.06	−0.39**	−0.31**	−0.32**	(0.88)

*N* = 420 responses (including 112 supervisors and 420 subordinates); Significant at: *p < 0.05; **p < 0.01; Figures in parentheses are alpha internal consistency reliabilities; OCB, Organizational citizenship behavior; PsyCap, Positive psychological capital.

*^1^Supervisor Gender = 1 = Male, 2 = Female;*

*^2^Supervisor Age = 1 = Less than 30 years, 2 = 31–35 years, 3 = 36–40 years, 4 = more than 40 years;*

*^3^Subordinate Gender = 1 = Male, 2 = Female;*

*^4^Subordinate Age = 1 = Less than 24 years, 2 = 25–28 years, 3 = 29–32 years, 4 = more than 32 years;*

*^5^Subordinate Education = 1 = High school, 2 = College education, 3 = Vocational education 4 = Others;*

*^6^Subordinate Working Experience in Service Industry = 1 = Less than 1 year, 2 = 1–3 years, 3 = 4–6 years, 4 = more than 6 years.*

### Test of Mediation

Table 2 presents the findings of the formal mediation test. OCB is positively correlated with ego depletion (*B* = 0.12, *SE* = 0.02, *t* = 5.64, *p* < 0.001, *LLCI* = 0.08, *ULCI* = 0.17) and service sabotage (*B* = 0.07, *SE* = 0.03, *t* = 2.71, *p* < 0.05, *LLCI* = 0.02, *ULCI* = 0.12). Ego depletion is positively correlated with service sabotage (*B* = 0.30, *SE* = 0.08, *t* = 3.91, *p* < 0.001, *LLCI* = 0.15, *ULCI* = 0.46) as well. Following the recent studies (e.g., see Tariq and Ding, 2018; Tariq and Weng, 2018; Tariq et al., 2020), we calculate bias-corrected bootstrapped confidence intervals (using 10,000 bootstrap samples) for indirect effects of OCB on service sabotage through ego depletion. Table 2 indicates the significant positive indirect effects of OCB on service sabotage through ego depletion (*B* = 0.04, *SE* = 0.01, *LLCI* = 0.02, *ULCI* = 0.06). Thus, the direct effects of OCB on service sabotage (*B* = 0.07, *SE* = 0.03, *LLCI* = 0.02, *ULCI* = 0.12), indirect effects of OCB on service sabotage via ego depletion (*B* = 0.04, *SE* = 0.01, *LLCI* = 0.02, *ULCI* = 0.06), and the total effects of OCB on service sabotage (*B* = 0.11, *SE* = 0.03, *LLCI* = 0.06, *ULCI* = 0.16) provide support for Hypothesis 1; that is, the positive relationship between OCB and service sabotage is mediated by ego depletion.

**TABLE 2:** Results of mediation analysis.

Antecedents	*Ego Depletion*						*Service Sabotage*					
		
	*B*	*SE*	*T*	*LLCI*	*ULCI*	*R*^2^	*B*	*SE*	*t*	*LLCI*	*ULCI*	*R*^2^
						0.18***						0.17***
Constant	3.90	0.23	16.66***	3.44	4.36		2.67	0.40	6.67***	1.88	3.45	
OCB	0.12	0.02	5.64***	0.08	–0.17		0.07	0.03	2.71*	0.02	0.12	
Ego Depletion	–	–	–	–	–		0.30	0.08	3.91***	0.15	0.46	
***Control Variables***												
Supervisor Gender	–0.06	0.06	–1.07	–0.17	0.05		0.12	0.06	1.92	0.00	0.24	
Supervisor Age	0.01	0.03	0.29	–0.06	0.08		0.03	0.04	0.88	–0.04	0.11	
Subordinate Gender	0.06	0.05	1.20	–0.04	0.16		–0.09	0.06	–1.56	–0.20	0.02	
Subordinate Age	0.01	0.02	0.46	–0.03	0.05		0.05	0.02	2.08	0.00	0.10	
Education	0.01	0.02	–0.18	–0.05	0.04		–0.02	0.03	–0.54	–0.07	0.04	
Experience in Service Industry	–0.05	0.02	–2.12	–0.09	0.00		0.01	0.03	0.57	–0.04	0.06	
Customers’ Negative Events	0.02	0.03	0.57	–0.04	0.07		–0.03	0.03	–0.96	–0.09	0.03	

**Predictor**				**Effect**			***SE***			**LLCI**		**ULCI**

*Direct effects*				
OCB on service sabotage				0.07			0.03			0.02		0.12
*Indirect effects*				
OCB on service sabotage via ego depletion				0.04			0.01			0.02		0.06
*Total effects*				
OCB on service sabotage				0.11			0.03			0.06		0.16

Results of direct, indirect, total, and normal theory effects of OCB on service sabotage. *N* = 420 responses (including 112 supervisors and 420 subordinates); Significant at: *p < 0.05; ***p < 0.001; LLCI, Lower level of the 95% confidence interval; ULCI, Upper level of 95% confidence interval; OCB, Organizational citizenship behavior.

### Test of the Moderated Mediation Model

**FIGURE 2:**
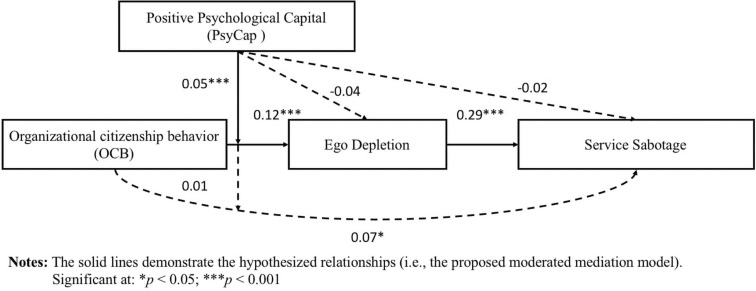
Results of moderated mediation model.

Table 3 and Figure 2 list the findings of our formal moderated mediation model. We found that OCB is positively correlated with ego depletion (*B* = 0.12, *SE* = 0.02, *t* = 5.72, *p* < 0.001, *LLCI* = 0.08, *ULCI* = 0.16) and service sabotage (*B* = 0.07, *SE* = 0.03, *t* = 2.68, *p* < 0.05, *LLCI* = 0.02, *ULCI* = 0.12). Ego depletion is also positively correlated with service sabotage (*B* = 0.29, *SE* = 0.08, *t* = 3.56, *p* < 0.001, *LLCI* = 0.13, *ULCI* = 0.44). The interaction term of OCB and PsyCap is negative and significant (*B* = −0.05, *SE* = 0.01, *t* = −3.60, *p* < 0.001, *LLCI* = −0.08, *ULCI* = −0.02), as indicated in Table 3. Thus, Hypothesis 2a is supported.

**TABLE 3:** Results of the moderated-mediation model analysis.

Antecedents	*Ego Depletion*						*Service Sabotage*					
		
	*B*	*SE*	*T*	*LLCI*	*ULCI*	*R*^2^	*B*	*SE*	*t*	*LLCI*	*ULCI*	*R*^2^
						0.23***						0.18***
Constant	4.37	0.21	20.43***	3.95	4.79		3.00	0.43	6.98***	2.15	3.85	
OCB	0.12	0.02	5.72***	0.08	0.16		0.07	0.03	2.68*	0.02	0.12	
Ego depletion	–	–	–	–	–		0.29	0.08	3.56***	0.13	0.44	
PsyCap	–0.04	0.02	–2.06	–0.07	0.00		–0.02	0.02	–0.82	–0.06	0.02	
OCB X PsyCap	–0.05	0.01	−3.60***	–0.08	–0.02		–0.01	0.02	–0.55	–0.04	0.02	
***Control Variables***												
Supervisor Gender	–0.05	0.05	–1.00	–0.16	0.05		0.12	0.06	1.95	0.00	0.25	
Supervisor Age	–0.01	0.03	–0.30	–0.08	0.06		0.03	0.04	0.72	–0.05	0.11	
Subordinate Gender	0.06	0.05	1.31	–0.03	0.16		–0.09	0.06	–1.51	–0.20	0.03	
Subordinate Age	0.01	0.02	0.11	–0.04	0.04		0.05	0.02	1.94	0.00	0.10	
Education	0.01	0.02	–0.11	–0.05	0.05		–0.01	0.03	–0.52	–0.07	0.04	
Experience in Service Industry	–0.05	0.02	–2.36	–0.09	–0.01		0.01	0.03	0.49	–0.04	0.06	
Customers’ Negative Events	0.03	0.03	1.16	–0.02	0.09		–0.02	0.03	–0.70	–0.09	0.04	

*N* = 420 responses (including 112 supervisors and 420 subordinates); Significant at: *p < 0.05; ***p < 0.001; LLCI, Lower level of the 95% confidence interval; ULCI, Upper level of 95% confidence interval; OCB, Organizational citizenship behavior; PsyCap, Positive psychological capital.

**FIGURE 3:**
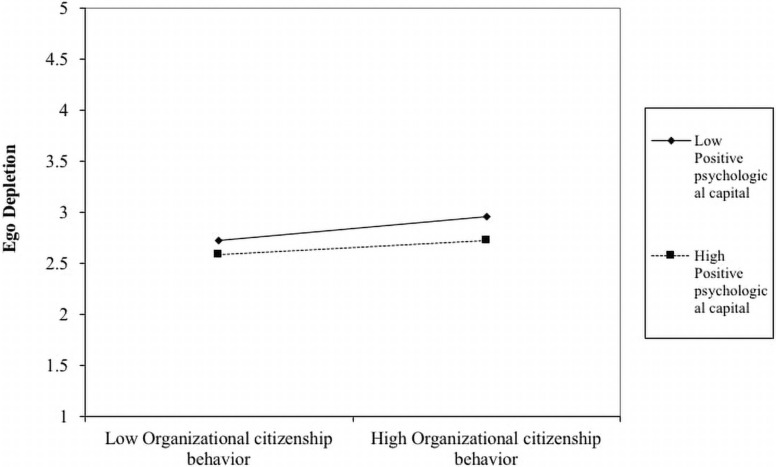
The interactive effect of OCB and Positive PsyCap on ego depletion.

Besides, we followed the work of Ahmad et al. (2019); Butt et al. (2019), Tariq et al. (2019), and Shillamkwese et al. (2019) to conduct the simple slop test to plot the moderating effect of PsyCap on the relationship between OCB and ego depletion. By doing so, we found that the relationship between OCB and ego depletion is stronger when PsyCap is low (*B* = 0.12, *t* = 2.68, *p* < 0.001) and weaker when PsyCap is high (*B* = 0.07, *t* = 1.67, *p* < 0.05). Thus, we again found support for Hypothesis 2a; that is, the positive relationship between OCB and ego depletion is moderated by PsyCap, such that this association is weaker when PsyCap is high. Also, to further support our Hypothesis 2(a), we plot the interaction term, i.e., organizational citizenship behavior X positive psychological capital, and provide the graphical presentation of the moderating effect of positive psychological capital. Figure 3 demonstrates that positive psychological capital moderates the positive relationship between organizational citizenship behavior and ego depletion, such that the positive relationship will be weaker when positive psychological capital is high.

To test Hypothesis 2b, we examined the conditional indirect effects of OCB on service sabotage via ego depletion at different values of PsyCap (−1 SD, M, and + 1 SD). Table 4 reveals that the indirect effect of OCB on service sabotage through ego depletion is weak when PsyCap is high (*B* = 0.01, *SE* = 0.01, *LLCI* = 0.01, *ULCI* = 0.04). This effect is strong when PsyCap is low (*B* = 0.06, *SE* = 0.02, *LLCI* = 0.02, *ULCI* = 0.09). In addition, the index of the moderated mediation is negative and significant (*effect* = −0.01, *SE* = 0.01, *LLCI* = −0.02, *ULCI* = −0.01). Therefore, our moderated mediation relationship (i.e., Hypothesis 2b) is supported; that is, the indirect positive relationship between OCB and service sabotage through ego depletion is moderated by PsyCap, such that the mediated relationship is weaker when PsyCap is high.

**TABLE 4:** Results of the conditional direct and indirect effects of OCB on service sabotage via ego depletion at values of PsyCap.

*Predictor*	*Mediator*	*Moderator*	*Effect*	*SE*	*LLCI*	*ULCI*
*Index of the moderated mediation model*	Ego depletion	PsyCap	–0.01	0.01	–0.02	–0.01
*Conditional direct effects*						
OCB on service sabotage	–	PsyCap at -1SD	0.08	0.04	0.01	0.16
OCB on service sabotage	–	PsyCap at Mean	0.07	0.03	0.02	0.12
OCB on service sabotage	–	PsyCap at + 1SD	0.06	0.03	–0.01	0.12
*Conditional indirect effects*						
OCB on service sabotage	Ego depletion	PsyCap at -1SD	0.06	0.02	0.02	0.09
OCB on service sabotage	Ego depletion	PsyCap at Mean	0.04	0.01	0.01	0.06
OCB on service sabotage	Ego depletion	PsyCap at + 1SD	0.01	0.01	0.01	0.04

**N* = 420 responses (including 112 supervisors and 420 subordinates); LLCI, Lower level of the 95% confidence interval; ULCI, Upper level of 95% confidence interval; OCB, Organizational citizenship behavior; PsyCap, Positive psychological capital.*

## Discussion

Though numerous studies (LePine et al., 2002; Turnipseed and Rassuli, 2005) have focused on the positive side of OCB for beneficiaries, this study aimed to highlight its potential cost by examining its immediate effect on its actors (those who exhibit OCB). Focusing on the dark side of OCB is important because exhibiting such behavior is common these days, which most likely has repercussions for its actors’ energy and other resources. This study began with two basic questions, “how OCB causes actors’ exhibition of service sabotage behavior through ego depletion” and “does actor’s PsyCap weakens the positive association between OCB and actors’ service sabotage behavior through ego depletion?” To answer these questions, we borrowed support from the ego-depletion theory. Traditionally, ego-depletion theory claims that work activities that involve attention on specific problems, resist directions, and manage attitudes all draw from the same pool of limited resources. Continuous self-regulation results in resource depletion, which renders the self temporarily less able and less willing to function normally or optimally. In the workplace, resource depletion leads to less effective self-regulation, which affects employees’ performance. For instance, Lin et al. (2016) stated that demanding interpersonal interactions deplete regulatory resources. Extrapolating this idea, this study proposed that OCB deplete resources owing to the demanding nature of this activity. Though several studies (Glasø et al., 2010; Chi et al., 2013) relied on an ego-depletion framework to demonstrate how it explains employees’ resource depletion and their failure to maintain set standards in the organization, how it explains the dark side of OCB by examining the relationship between OCB and employees’ service sabotage behavior is ignored to date. To fill this gap, this study provides a mechanism to answer our first question, i.e., how an exhibition of OCB results in actors’ involvement in service sabotage behavior. This study proposes that when employees exhibit OCB, it consumes more of their available regulatory resources, leading to feeling depleted. In such circumstances, where employees are left with limited regulatory resources, the depleted employees not only are unable to perform their extra-role performance (OCB) but are also unable to perform assigned role performance. Specifically, we found that when employees exhibit OCB, it depletes their pool of resources, which affects the service standards (service sabotage). The findings of this study are in line with prior literature (Hagger et al., 2010; Carter et al., 2015; Dang, 2018) which documented that depleted employees lack sufficient resources to maintain their productive behavior and to perform appropriately at the workplace.

Besides, this study also demonstrates that OCB may not always result in undesired behavior on the actors’ part, which directly answers our second question; i.e., does the actors’ PsyCap weaken the positive association between OCB and service sabotage behavior through ego depletion? As hypothesized, our findings show that PsyCap weakens the direct positive association between OCB and ego depletion but weakens the indirect relationship between OCB and service sabotage; i.e., detrimental effects of OCB are lower for those who are high in PsyCap. Our findings reveal that when employees exhibit OCB, their resource pool depletes, which affects the normal functioning of their regulatory system. In such a scenario, the availability of PsyCap enables employees to offset the resource loss caused by OCB, and thus, employees’ regulatory system works properly. Specifically, the study finds that PsyCap compensates for resource loss and helps employees to maintain their service standards.

This study took a novel perspective on the effect of OCB by arguing theoretically and providing empirical support to the fact that when employees engage in OCB, they experience resource depletion. From a theoretical perspective, we contribute to the ego-depletion literature by identifying a phenomenon that consumes resources. Going beyond existing research is mainly preoccupied with identifying those factors that just drain resources. At the same time, less attention has been devoted to identifying such factors that provide resources or maintain the available resources. This study investigated the impact of employees’ OCB as a phenomenon that depletes resources. While OCB comes with an immediate cost for its actors, it also causes adverse effects for organizations. Besides pointing out OCB as a resource-consuming phenomenon, this study also incorporated PsyCap as a resource-replenishing factor and examined its effect in dumping the negative effect of OCB. While OCB comes at some cost, this study also highlighted some bright side to this story: resources are replenished when employees have higher PsyCap.

Given the effect of OCB, the findings of this study have important implications for research on PsyCap. Specifically, our findings suggest that PsyCap provides the necessary resources to invest in an important yet depleting activity that may render resource depletion for actors. In the context of OCB, it shows that high PsyCap exacerbated the depleting effect of OCB. Arguably, it happens because employees with high PsyCap have sufficient resources that generate prosocial motivation. Such employees are preoccupied with exhibiting OCB and may therefore not experience that much depletion compared to employees with lower PsyCap, which later restricts their service sabotage behavior. Moving beyond PsyCap, this study also contributes to research on the association between OCB and service sabotage by highlighting the complex mechanism of ego depletion. Consistent with ego-depletion theory, this study suggested that OCB consumes employees’ regulatory resources, which decreases their ability to perform their tasks. Specifically, our findings suggest that those employees who exhibit OCB experience ego depletion, which affects their service quality (service sabotage).

The findings of this study also offer practical implications for employees and organizations. Specifically, the findings suggest that employees ought to exercise caution while exhibiting OCB, because such behavior may leave them depleted and less effective at their assigned tasks. Also, our findings carry implications for organizations seeking OCB from employees. First, organizations need to realize that expecting such extra-role task has detrimental effects for exhibitors. This is not to say that organizations should not encourage employees’ OCB behavior but that the organizations must compensate those employees who go beyond their assigned task and perform extra-role tasks. Moreover, grounding on our findings, i.e., PsyCap weakens the adverse effect of OCB and reduces employees’ service sabotage behavior, this study provides important insights into various human resource practices, such as the selection of candidates and training workshops for PsyCap. For example, organizations could access applicants’ level of PsyCap during the selection process through some standard written tests. By utilizing certain Psychological Capital Questionnaire tests, organizations could hire a job candidate with positive personal qualities. As suggested, a key feature of PsyCap is that it is malleable and could be developed (Luthans et al., 2007b), so we suggest organizations to enhance their employees’ PsyCap by providing them sufficient training so they could perform better even in depleted circumstances.

### Limitations

We believe that this study opens new doors for future researchers in OCB literature and further highlights its dark side. But we also accept that besides the interesting findings, this study has several limitations which need to be mentioned here. First, this study considers OCB a general phenomenon; i.e., it does not consider OCB dimensions: OCBI and OCBO; it is quite possible that employees exhibiting OCBI do not exhibit OCBO. To better understand the phenomenon (OCB–service sabotage), it would be better to examine both dimensions separately. Further, this study did not consider specific OCB episodes often performed by employees due to survey length and time constraints. This is another limitation of this study because the nature of OCB tasks may impact the effect of OCB on ego depletion. For instance, helping others with some novel problems may require extra resources which cause more ego depletion compared to helping others in routine matters. Similarly, extending OCB toward a well-liked coworker may cause less depletion compared to others. Following the prior literature, this study controlled some demographic factors and customers’ negative events, potentially affecting our proposed relationship. However, there may be some other factors that we did not control, but they could potentially inflate our findings. For instance, if an employee experiences work–family conflict (WFC) or any other stress at the workplace, it would also deplete his/her resources, leading to service sabotage. Here, we encourage future researchers to consider such factors while examining the same related phenomenon. Fourth, though the findings are based on time-lagged multi-source data, we cannot eliminate the potential issue of common method bias. It might be possible that employees exhibiting OCB might not get depleted immediately but may get depleted after a certain period. For that, it is recommended to adopt a time series approach or a daily diary method. Fifth, based on an ego-depletion framework, this study examined the relationship between OCB and service sabotage through ego depletion; however, it is not the only mechanism that explains this relationship. Future researchers are encouraged to incorporate other mechanisms (e.g., moral credits) which may better explain the relationships. Fifth, the findings are based on only one industry in China; one should consider the generalizability issue across other industries and cultures.

Finally, in our study, we only hypothesized and focused on the relationship between OCB and service sabotage via ego depletion at high PsyCap. The simple slope test and the moderated mediation results demonstrate that the indirect effects of OCB on service sabotage via ego depletion is weaker (stronger) when PsyCap is high (low). Therefore, we call for further studies to integrate the relevant theory to hypothesize the indirect effect on high vs. low values of PsyCap and to conduct empirical tests to provide more understanding about the relationship of OCB and service sabotage via ego depletion at high vs. low values of PsyCap.

Despite certain limitations, we hope that future researchers build upon our findings and further explore the dark side of OCB. Although being involved in OCB is critical behavior at the workplace, it is rational to establish a balanced view of its cost and benefits. The ideal situation, after all, is for employees to exhibit OCB without depleting their own will.

## Conclusion

Despite the most important extra-role expectations from employees, OCB has some immediate cost for those who exhibit it and later for the organizations and all those directly or indirectly associated with employees or organizations. This study contributes to the existing literature on OCB and service sabotage by providing a mechanism that explains how most desired behavior becomes costly for the organizations, i.e., how OCB leads to employees’ service sabotage behavior through ego depletion. Besides, it also answers how employees with high PsyCap experience and behave after exhibiting OCB. This study concludes that when employees exhibit OCB, their limited pool of resources is depleted, restricting their normal behavior. In contrast, PsyCap provides employees with more resources to compensate for their resource loss, leading them to become less depleted and have less service sabotage behavior. We sincerely hope that other researchers will join us in highlighting other costs associated with OCB.

## Data Availability Statement

The raw data supporting the conclusions of this article will be made available by the authors, without undue reservation.

## Ethics Statement

The studies involving human participants were reviewed and approved by the Ethics Committee at Jiangsu University. The patients/participants provided their written informed consent to participate in this study.

## Author Contributions

All authors equally contributed to conception and design, acquisition of data, analysis and interpretation of data. They also drafted the article for important intellectual content. All authors approved final version to be published and agreement to be accountable for all aspects of the work in ensuring that questions related to the accuracy or integrity of any part of the work are appropriately investigated and resolved.

## Informed Consent

The informed consent was obtained verbally as well as in writing from the participants. A cover letter was shared with them that guaranteed the confidentiality of their responses. The participation of the respondents was voluntary. The purpose of the study was also shared with them. The participants were encouraged to stay in contact with the researcher through the provided contact information.

## Conflict of Interest

The authors declare that the research was conducted in the absence of any commercial or financial relationships that could be construed as a potential conflict of interest.

## References

[B1] Ahmad B. Tariq H. Weng Q. Shillamkwese Samson S. Sohail N. (2019). When a proximate starts to gossip: instrumentality considerations in the emergence of abusive supervision. *Employee Relat. Int. J.* 41 851–875. 10.1108/er-08-2018-0225

[B2] Avey J. B. Avolio B. J. Luthans F. (2011). Experimentally analyzing the impact of leader positivity on follower positivity and performance. *Leadersh. Q.* 22 282–294. 10.1016/j.leaqua.2011.02.004

[B3] Avey J. B. Luthans F. Smith R. M. Palmer N. F. (2010a). Impact of positive psychological capital on employee wellbeing over time. *J. Occup. Health Psychol.* 15:17. 10.1037/a0016998 20063956

[B4] Avey J. B. Nimnicht J. L. Graber Pigeon N. (2010b). Two field studies examining the association between positive psychological capital and employee performance. *Leadersh. Organ. Dev. J.* 31 384–401. 10.1108/01437731011056425

[B5] Avey J. B. Wernsing T. S. Luthans F. (2008). Can positive employees help positive organizational change? Impact of psychological capital and emotions on relevant attitudes and behaviors. *J. Appl. Behav. Sci.* 44 48–70. 10.1177/0021886307311470

[B6] Babcock-Roberson M. E. Strickland O. J. (2010). The relationship between charismatic leadership, work engagement, and organizational citizenship behaviors. *J. Psychol.* 144 313–326. 10.1080/00223981003648336 20461933

[B7] Bakker A. B. Demerouti E. (2007). The job demands-resources model: state of the art. *J. Manag. Psychol.* 22 309–328. 10.1108/02683940710733115

[B8] Bandura A. (1997). *Self-Efficacy: The Exercise of Control.* New York, NY: Macmillan.

[B9] Barnes C. M. Lucianetti L. Bhave D. P. Christian M. S. (2015). “You wouldn’t like me when I’m sleepy”: leaders’ sleep, daily abusive supervision, and work unit engagement. *Acad. Manag. J.* 58 1419–1437. 10.5465/amj.2013.1063

[B10] Bateman T. S. Organ D. W. (1983). Job satisfaction and the good soldier: the relationship between affect and employee “citizenship”. *Acad. Manag. J.* 26 587–595. 10.5465/255908

[B11] Bauer I. M. Baumeister R. F. (2011). Self-regulatory strength. *Handb. Self Regul. Res. Theory Appl.* 2 64–82.

[B12] Baumeister R. E. Bratslavsky E. Muraven M. Tice D. M. (1998). Ego depletion: is the active self a limited resource? *J. Pers. Soc. Psychol.* 74 1252–1265. 10.1037/0022-3514.74.5.1252 9599441

[B13] Baumeister R. F. Bratslavsky E. Muraven M. (2018). *Ego depletion: Is the active self a limited resource? Self-Regulation and Self-Control.* London: Routledge, 24–52. 10.1037//0022-3514.74.5.1252 9599441

[B14] Baumeister R. F. Leary M. R. (1995). The need to belong: desire for interpersonal attachments as a fundamental human motivation. *Psychol. Bull.* 117:497. 10.1037/0033-2909.117.3.497 7777651

[B15] Bell S. J. Menguc B. (2002). The employee-organization relationship, organizational citizenship behaviors, and superior service quality. *J. Retail.* 78 131–146. 10.1016/s0022-4359(02)00069-6

[B16] Bergeron D. M. (2007). The potential paradox of organizational citizenship behavior: good citizens at what cost? *Acad. Manag. Rev.* 32 1078–1095. 10.5465/amr.2007.26585791

[B17] Bienstock C. C. DeMoranville C. W. Smith R. K. (2003). Organizational citizenship behavior and service quality. *J. Serv. Mark.* 17 357–378.

[B18] Bolino M. C. Turnley W. H. (2003). Going the extra mile: cultivating and managing employee citizenship behavior. *Acad. Manag. Perspect.* 17 60–71. 10.5465/ame.2003.10954754

[B19] Bolino M. C. Turnley W. H. (2005). The personal costs of citizenship behavior: the relationship between individual initiative and role overload, job stress, and work-family conflict. *J. Appl. Psychol.* 90:740. 10.1037/0021-9010.90.4.740 16060790

[B20] Brislin R. W. (1980). Translation and content analysis of oral and written material. *Handb. Cross Cult. Psychol.* 2 349–444.

[B21] Butt H. P. Tariq H. Weng Q. Sohail N. (2019). I see you in me, and me in you: the moderated mediation crossover model of work passion. *Pers. Rev.* 48 1209–1238. 10.1108/PR-05-2018-0176

[B22] Carter E. C. Kofler L. M. Forster D. E. McCullough M. E. (2015). A series of meta-analytic tests of the depletion effect: self-control does not seem to rely on a limited resource. *J. Exp. Psychol. Gen.* 144:796. 10.1037/xge0000083 26076043

[B23] Chi N.-W. Tsai W.-C. Tseng S.-M. (2013). Customer negative events and employee service sabotage: the roles of employee hostility, personality and group affective tone. *Work Stress* 27 298–319. 10.1080/02678373.2013.819046

[B24] Dang J. (2018). An updated meta-analysis of the ego depletion effect. *Psychol. Res.* 82 645–651. 10.1007/s00426-017-0862-x 28391367 PMC6013521

[B25] Dang J. Barker P. Baumert A. Bentvelzen M. Berkman E. Buchholz N. (2020). A multilab replication of the ego depletion effect. *Soc. Psychol. Pers. Sci.* [Epub ahead of print]. 10.1177/1948550619887702 PMC8186735 34113424

[B26] Daniels D. Joireman J. Falvy J. Kamdar D. (2006). Organizational citizenship behavior as function of empathy consideration of future consequences, and employee time horizon: an initial exploration using an in-basket simulation of OCBs. *J. Appl. Soc. Psychol.* 36 2266–2292. 10.1111/j.0021-9029.2006.00103.x

[B27] Deery S. Rayton B. Walsh J. Kinnie N. (2017). The costs of exhibiting organizational citizenship behavior. *Hum. Resour. Manag.* 56 1039–1049. 10.1002/hrm.21815

[B28] Duffy M. K. Scott K. L. Shaw J. D. Tepper B. J. Aquino K. (2012). A social context model of envy and social undermining. *Acad. Manag. J.* 55 643–666. 10.5465/amj.2009.0804

[B29] Edwards J. R. Lambert L. S. (2007). Methods for integrating moderation and mediation: a general analytical framework using moderated path analysis. *Psychol. Methods* 12 1–22. 10.1037/1082-989x.12.1.1 17402809

[B30] Eissa G. Lester S. W. (2017). Supervisor role overload and frustration as antecedents of abusive supervision: the moderating role of supervisor personality. *J. Organ. Behav.* 38 307–326. 10.1002/job.2123

[B31] Fishbach A. Labroo A. A. (2007). Be better or be merry: how mood affects self-control. *J. Pers. Soc. Psychol.* 93:158. 10.1037/0022-3514.93.2.158 17645393

[B32] Friese M. Loschelder D. D. Gieseler K. Frankenbach J. Inzlicht M. (2019). Is ego depletion real? An analysis of arguments. *Pers. Soc. Psychol. Rev.* 23 107–131. 10.1177/1088868318762183 29591537

[B33] Glasø L. Vie T. L. Holmdal G. R. Einarsen S. (2010). An application of affective events theory to workplace bullying. *Eur. Psychol.* 16 198–208. 10.1027/1016-9040/a000026

[B34] Gyekye S. A. Haybatollahi M. (2015). Organizational citizenship behaviour: an empirical investigation of the impact of age and job satisfaction on Ghanaian industrial workers. *Int. J. Organ. Anal.* 23 285–301. 10.1108/ijoa-08-2012-0586

[B35] Hagger M. S. Wood C. Stiff C. Chatzisarantis N. L. (2010). Ego depletion and the strength model of self-control: a meta-analysis. *Psychol. Bull.* 136:495. 10.1037/a0019486 20565167

[B36] Harper D. (1990). Spotlight abuse-save profits. *Ind. Distrib.* 79 47–51.

[B37] Harris L. C. Ogbonna E. (2002). Exploring service sabotage: the antecedents, types and consequences of frontline, deviant, antiservice behaviors. *J. Serv. Res.* 4 163–183. 10.1177/1094670502004003001

[B38] Harris L. C. Ogbonna E. (2006). Service sabotage: a study of antecedents and consequences. *J. Acad. Mark. Sci.* 34 543–558. 10.1177/0092070306287324

[B39] Harris L. C. Ogbonna E. (2009). Service sabotage: the dark side of service dynamics. *Bus. Horizons* 52 325–335. 10.1016/j.bushor.2009.02.003

[B40] Harris L. C. Ogbonna E. (2012). Motives for service sabotage: an empirical study of frontline workers. *Serv. Ind. J.* 32 2027–2046. 10.1080/02642069.2011.582496

[B41] Hayes A. F. (2013). *Methodology in The Social Sciences: Introduction to Mediation, Moderation, and Conditional Process Analysis: A Regression-Based Approach.* New York, NY: Guilford Press.

[B42] Hongbo L. Waqas M. Tariq H. (2019). From victim to saboteur: testing a moderated mediation model of perceived undermining, state hostility, and service sabotage. *J. Serv. Theory Pract.* 29 2–21. 10.1108/JSTP-02-2018-0030

[B43] Hongbo L. Waqas M. Tariq H. Abena A. A. N. Akwasi O. C. Ashraf S. F. (2020). I will hurt you for this, when and how subordinates take revenge from abusive supervisors: a perspective of displaced revenge. *Front. Psychol*. 11:503153. 10.3389/fpsyg.2020.503153 33101111 PMC7546874

[B44] Indarti S. Fernandes A. A. R. Hakim W. (2017). The effect of OCB in relationship between personality, organizational commitment and job satisfaction on performance. *J. Manag. Dev.* 36 1283–1293. 10.1108/jmd-11-2016-0250

[B45] Jafri H. (2012). Psychological capital and innovative behaviour: an empirical study on apparel fashion industry. *J. Contemp. Manag. Res.* 2 42–52.

[B46] Jermier J. (1988). Sabotage at work: the rational view. *Res. Sociol. Organ.* 6:34.

[B47] Kao F. H. Cheng B. S. Kuo C. C. Huang M. P. (2014). Stressors, withdrawal, and sabotage in frontline employees: the moderating effects of caring and service climates. *J. Occup. Organ. Psychol.* 87 755–780. 10.1111/joop.12073

[B48] Karatepe O. M. Karadas G. (2015). Do psychological capital and work engagement foster frontline employees’ satisfaction? A study in the hotel industry. *Int. J. Contemp. Hosp. Manag.* 27 1254–1278. 10.1108/ijchm-01-2014-0028

[B49] Kenny D. A. (1995). The effect of nonindependence on significance testing in dyadic research. Personal relationships, 2, 67–75. 10.1111/j.1475-6811.1995.tb00078.x

[B50] Lanaj K. Johnson R. E. Wang M. (2016). When lending a hand depletes the will: the daily costs and benefits of helping. *J. Appl. Psychol.* 101:1097. 10.1037/apl0000118 27149605

[B51] Latif K. Tariq H. Khan A. K. Weng Q. Butt H. P. Obaid A. (2020). Loaded with knowledge, yet green with envy: leader-member exchange comparison and coworkers-directed knowledge hiding behavior. *J. Knowl. Manag.* 24, 1653–1680. 10.1108/JKM-10-2019-0534

[B52] Lavy S. Littman-Ovadia H. (2017). My better self: using strengths at work and work productivity, organizational citizenship behavior, and satisfaction. *J. Career Dev.* 44 95–109. 10.1177/0894845316634056

[B53] Lee A. Gerbasi A. Schwarz G. Newman A. (2019). Leader–member exchange social comparisons and follower outcomes: the roles of felt obligation and psychological entitlement. *J. Occup. Organ. Psychol.* 92 593–617. 10.1111/joop.12245

[B54] Lee J. J. Ok C. M. (2014). Understanding hotel employees’ service sabotage: emotional labor perspective based on conservation of resources theory. *Int. J. Hosp. Manag.* 36 176–187. 10.1016/j.ijhm.2013.08.014

[B55] Lee K. Allen N. J. (2002). Organizational citizenship behavior and workplace deviance: the role of affect and cognitions. *J. Appl. Psychol.* 87:131. 10.1037/0021-9010.87.1.131 11916207

[B56] LePine J. A. Erez A. Johnson D. E. (2002). The nature and dimensionality of organizational citizenship behavior: a critical review and meta-analysis. *J. Appl. Psychol.* 87:52. 10.1037/0021-9010.87.1.52 11916216

[B57] Lin S.-H. J. Ma J. Johnson R. E. (2016). When ethical leader behavior breaks bad: how ethical leader behavior can turn abusive via ego depletion and moral licensing. *J. Appl. Psychol.* 101 815–830. 10.1037/apl0000098 26867103

[B58] Luthans F. Avey J. B. Avolio B. J. Peterson S. J. (2010). The development and resulting performance impact of positive psychological capital. *Hum. Resour. Dev. Q.* 21 41–67. 10.1002/hrdq.20034

[B59] Luthans F. Avolio B. J. Avey J. B. Norman S. M. (2007). Positive psychological capital: measurement and relationship with performance and satisfaction. *Pers. Psychol.* 60 541–572. 10.1111/j.1744-6570.2007.00083.x

[B60] Luthans F. Norman S. M. Avolio B. J. Avey J. B. (2008). The mediating role of psychological capital in the supportive organizational climate—employee performance relationship. *J. Organ. Behav.* 29 219–238. 10.1002/job.507

[B61] Luthans F. Youssef C. M. Avolio B. J. (2007a). *Psychological Capital: Developing the Human Competitive Edge.* Oxford: Oxford University Press.

[B62] Luthans F. Youssef C. M. Avolio B. J. (2007b). Psychological capital: investing and developing positive organizational behavior. *Posit. Organ. Behav.* 1 9–24. 10.4135/9781446212752.n2

[B63] Morrison E. W. (1996). Organizational citizenship behavior as a critical link between HRM practices and service quality. *Hum. Resour. Manag.* 35 493–512. 10.1002/(sici)1099-050x(199624)35:4<493::aid-hrm4>3.0.co;2-r

[B64] Organ D. W. (1990). The motivational basis of organizational citizenship behavior. *Res. Organ. Behav.* 12 43–72.

[B65] Organ D. W. (1997). Organizational citizenship behavior: it’s construct clean-up time. *Hum. Perform.* 10 85–97. 10.1207/s15327043hup1002_2

[B66] Organ D. W. Ryan K. (1995). A meta-analytic review of attitudinal and dispositional predictors of organizational citizenship behavior. *Pers. Psychol.* 48 775–802. 10.1111/j.1744-6570.1995.tb01781.x

[B67] Preacher K. J. Rucker D. D. Hayes A. F. (2007). Addressing moderated mediation hypotheses: theory, methods, and prescriptions. *Multiv. Behav. Res.* 42 185–227. 10.1080/00273170701341316 26821081

[B68] Seligman M. E. (2000). *dan Csikszentmihalyi, Mihaly. Positive Psychology: An Introduction.* Washington, DC: American Psychological Association, 5–14. 10.1037//0003-066x.55.1.5 11392865

[B69] Settoon R. P. Mossholder K. W. (2002). Relationship quality and relationship context as antecedents of person-and task-focused interpersonal citizenship behavior. *J. Appl. Psychol.* 87 255–267. 10.1037/0021-9010.87.2.255 12002954

[B70] Shillamkwese S. S. Tariq H. Obaid A. Weng Q. Garavan T. N. (2019). It’s not me, it’s you: testing a moderated mediation model of subordinate deviance and abusive supervision through the self-regulatory perspective. *Bus. Ethics Eur. Rev.* 29 227–243. 10.1111/beer.12245

[B71] Skarlicki D. P. Van Jaarsveld D. D. Walker D. D. (2008). Getting even for customer mistreatment: the role of moral identity in the relationship between customer interpersonal injustice and employee sabotage. *J. Appl. Psychol.* 93 1335–1347. 10.1037/a0012704 19025251

[B72] Smith C. Organ D. W. Near J. P. (1983). Organizational citizenship behavior: its nature and antecedents. *J. Appl. Psychol.* 68 653–663.

[B73] Stajkovic A. D. Luthans F. (1998). Social cognitive theory and self-efficacy: goin beyond traditional motivational and behavioral approaches. *Organ. Dyn.* 26 62–74. 10.1016/s0090-2616(98)90006-7

[B74] Steel P. (2007). The nature of procrastination: a meta-analytic and theoretical review of quintessential self-regulatory failure. *Psychol. Bull.* 133 65–94. 10.1037/0033-2909.133.1.65 17201571

[B75] Tao C. W. W. Jang J. Kwon J. (2019). Understanding the role of emotional intelligence and work status in service sabotage: Developing and testing a three-way interaction model. *J. Hosp. Tour. Manag.* 41, 51–59. 10.1016/j.jhtm.2019.09.005Get

[B76] Tariq H. Ding D. (2018). Why am I still doing this job? The examination of family motivation on employees’ work behaviors under abusive supervision. *Pers. Rev.* 47 378–402. 10.1108/pr-07-2016-0162

[B77] Tariq H. Weng Q. (2018). Accountability breeds response-ability: instrumental contemplation of abusive supervision. *Pers. Rev.* 47 1019–1042. 10.1108/pr-05-2017-0149

[B78] Tariq H. Weng Q. Garavan T. N. Obaid A. Hassan W. (2020). Another sleepless night: does a leader’s poor sleep lead to subordinate’s poor sleep? A spillover/crossover perspective. *J. Sleep Res.* 29:e12904. 10.1111/jsr.12904 31578789

[B79] Tariq H. Weng Q. Ilies R. Khan A. K. (2019). Supervisory abuse of high performers: a social comparison perspective. *Appl. Psychol.* 70 280–310. 10.1111/apps.12229

[B80] Turnipseed D. L. Rassuli A. (2005). Performance perceptions of organizational citizenship behaviours at work: a bi-level study among managers and employees. *Br. J. Manag.* 16 231–244. 10.1111/j.1467-8551.2005.00456.x

[B81] Twenge J. Muraven M. Tice D. (2004). *Measuring State Self-Control: Reliability, Validity, and Correlations with Physical and Psychological Stress.* San Diego, CA: San Diego State University.

[B82] Weng Q. Butt H. P. Almeida S. Ahmed B. Obaid A. Burhan M. (2020). Where energy flows, passion grows: testing a moderated mediation model of work passion through a cross-cultural lens. *Curr. Psychol.* 1–15. 10.1007/s12144-020-01071-x

[B83] Yam K. C. Chen X.-P. Reynolds S. J. (2014). Ego depletion and its paradoxical effects on ethical decision making. *Organ. Behav. Hum. Dec. Process.* 124 204–214. 10.1016/j.obhdp.2014.03.008

[B84] Yeh C.-W. (2015). Linking customer verbal aggression and service sabotage. *J. Serv. Theory Pract.* 25 877–896. 10.1108/jstp-07-2014-0146

